# Antimicrobial Activity of an Fmoc-Plantaricin 149 Derivative Peptide against Multidrug-Resistant Bacteria

**DOI:** 10.3390/antibiotics12020391

**Published:** 2023-02-15

**Authors:** Gabriela Marinho Righetto, José Luiz de Souza Lopes, Paulo José Martins Bispo, Camille André, Julia Medeiros Souza, Adriano Defini Andricopulo, Leila Maria Beltramini, Ilana Lopes Baratella da Cunha Camargo

**Affiliations:** 1 Laboratory of Molecular Epidemiology and Microbiology, Department of Physics and Interdisciplinary Science, São Carlos Institute of Physics, University of São Paulo, São Carlos 13563-120, Brazil; 2 Laboratory of Applied Biophysics, Department of Applied Physics, Institute of Physics, University of São Paulo, São Paulo 05315-970, Brazil; 3 Department of Ophthalmology, Infectious Disease Institute, Massachusetts Eye and Ear, Harvard Medical School, Boston, MA 02115, USA; 4 Laboratory of Medicinal and Computational Chemistry, Department of Physics and Interdisciplinary Science, São Carlos Institute of Physics, University of São Paulo, São Carlos 13563-120, Brazil; 5 Group of Biophysics and Structural Biology “Sérgio Mascarenhas”, Department of Physics and Interdisciplinary Science, São Carlos Institute of Physics, University of São Paulo, São Carlos 13563-120, Brazil

**Keywords:** Plantaricin 149, synergism, carpet-like mechanism

## Abstract

Antimicrobial resistance poses a major threat to public health. Given the paucity of novel antimicrobials to treat resistant infections, the emergence of multidrug-resistant bacteria renewed interest in antimicrobial peptides as potential therapeutics. This study designed a new analog of the antimicrobial peptide Plantaricin 149 (Pln149-PEP20) based on previous Fmoc-peptides. The minimal inhibitory concentrations of Pln149-PEP20 were determined for 60 bacteria of different species and resistance profiles, ranging from 1 mg/L to 128 mg/L for Gram-positive bacteria and 16 to 512 mg/L for Gram-negative. Furthermore, Pln149-PEP20 demonstrated excellent bactericidal activity within one hour. To determine the propensity to develop resistance to Pln149-PEP20, a directed-evolution in vitro experiment was performed. Whole-genome sequencing of selected mutants with increased MICs and wild-type isolates revealed that most mutations were concentrated in genes associated with membrane metabolism, indicating the most likely target of Pln149-PEP20. Synchrotron radiation circular dichroism showed how this molecule disturbs the membranes, suggesting a carpet mode of interaction. Membrane depolarization and transmission electron microscopy assays supported these two hypotheses, although a secondary intracellular mechanism of action is possible. The molecule studied in this research has the potential to be used as a novel antimicrobial therapy, although further modifications and optimization remain possible.

## 1. Introduction

Antimicrobial resistance can occur naturally over time, typically through genetic changes. The process is, therefore, natural and inevitable [1]. However, the exponential increase in antimicrobial resistance has led to the endemic progression of infections and multidrug-resistant (MDR) phenotypes [2].

The antibiotics currently available for treating common infections are often inefficient. Therefore, alternative treatments must be identified [3]. Antimicrobial peptides (AMPs) represent an attractive option because they usually present a broad spectrum of activity, produce immunomodulatory effects, and can ward off resistance [4]. AMPs are now a small but growing category in the market. The antimicrobial peptide database contains more than 3400 molecules of different categories and biological sources [5]. Currently, a few AMPs are used mainly as a “last resource” treatment, for example, gramicidin, polymyxin, and daptomycin, while some are in clinical and pre-clinical evaluation, for example, nisin and LL-37 cathelicidin [6].

*Lactobacillus plantarum*, a Gram-positive bacterium widely distributed in nature, produces bacteriocins called “plantaricins”. Bacteriocin peptides are an essential group of AMPs, synthesized by bacterial ribosomes, which interfere with the growth of other microorganisms for protection. Plantaricin A (PlnA), a 26-aminoacid length cationic peptide with membrane-permeabilizing properties, is one of the best-characterized members of this group [7]. Plantaricin 149 (Pln149), a second member of this group, obtained from the strain *L. plantarum* NRIC 149, shares the same N-terminal sequence as PlnA. Pln149 contains the sequence YSLQMGATAIKQVKKLFKKKGG and shows inhibitory activity against other lactobacilli. Its synthetic version, a C-terminal amidated peptide, showed meaningful action against *S. aureus* and *Listeria monocytogenes* [8].

Like PlnA, the synthetic analogs of Pln149 preserved their activity against pathogenic bacteria, even after removing the N-terminal pentapeptide [9]. The typical disorder-to-helix conformational changes observed in many linear cationic AMPs are also maintained. Attempts to optimize Pln149 included keeping the fluorenylmethyloxycarbonyl (Fmoc) protecting group in the N-terminal portion of the AMP. This change yielded significant, hitherto-unobserved findings, such as an improved action on Gram-positive bacteria, and promoted action on gram-negative bacteria [9]. The uptake of the Fmoc group is thought to be the reason for its more significant action in Gram-negative bacteria, which have an outer membrane absent in Gram-positive bacteria. In contrast, the solid hydrophobic action of Fmoc led to increased toxicity because it increased the interaction with zwitterionic phospholipids.

Subsequently, the physicochemical mechanisms that occur during the interaction between the peptide and membrane models were elucidated [10]. This interaction depends on the negative electron charge density of the membrane, and can cause a disruptive effect. Adsorption on zwitterionic membranes has also been observed, which explains the action described in *Saccharomyces cerevisiae*. Factors other than charge seem to modulate the action of Pln149, such as curvature, lipid organization, and degree of hydration [11,12]. These studies suggested that Pln149 acts carpet-like, in which the helices align parallel to the membrane surface [10,13].

In this study, we present Pln149-PEP20 (Fmoc-KAVKKLFKKWG), an optimized molecule based on Fmoc-Pln149(6-22), which was designed as an amphipathic alpha helix to enhance antimicrobial activity and lower cytotoxicity. *Enterococcus faecium*, *Staphylococcus aureus*, *Klebsiella pneumoniae*, *Acinetobacter baumannii*, *Pseudomonas aeruginosa*, and *Enterobacter* spp. (ESKAPE) pathogenic bacteria were used as study models. ESKAPE bacteria are associated with the highest burden of MDR infections worldwide [14]. While ESKAPE is an acronym, the term also refers to the bacteria’s ability to escape antimicrobial action and cause MDR infections that are refractory to most first-line antibiotics. The WHO lists these organisms as priority pathogens that pose the greatest threat to human and public health, for which the development of new antimicrobials is urgently required [15].

## 2. Results

### 2.1. Quantitative Peptide Comparison and Antimicrobial Susceptibility

The antimicrobial activities of the new derivative peptides, Fmoc-Pln149(6-22) and Fmoc-Pln149, as determined by their MIC and MBC against Gram-positive and Gram-negative bacteria, were compared (Table 1).

In evaluating the hemolytic activity of Fmoc-Pln149, the high toxicity appeared to be a direct consequence of the presence of the Fmoc-protecting group, as it has a highly hydrophobic presence that could lead to interactions with the zwitterionic phospholipids on the eukaryotic membrane. Upon deeper investigation, the evaluation of the other two peptides, Fmoc-Pln149(6-22) and Pln149-PEP20, showed that Fmoc is not the primary influence on hemolytic activity. This observation is based on the fact that it was possible to drastically decrease hemolytic activity by modifying the size and sequence of amino acids.

Finally, the proposed peptide was evaluated against more than 60 strains. It was found to be active against all, with MICs ranging from 1 mg/L to 128 mg/L for Gram-positive bacteria and 16 mg/L to 512 mg/L for Gram-negative bacteria (Appendix A, Table A1 and Table A2). Pln149-PEP20 is active against strains with varying antimicrobial susceptibility profiles. Resistance mechanisms to different antibiotics, such as tigecycline, oxacillin, vancomycin, and daptomycin, did not affect the activity of the peptide.

### 2.2. Time-Kill and Post-Antibiotic Assays

**Figure 1:**
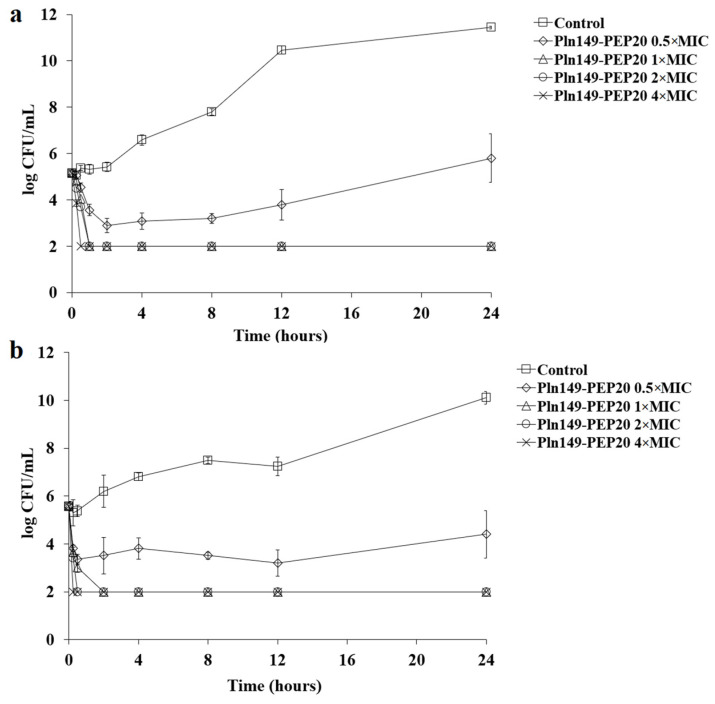
Pln149-PEP20 time-kill against (**a**) *S. aureus* ATCC 25923 (MIC = 8 mg/L) and (**b**) *S. aureus* SA43 (MIC = 8 mg/L).

**Figure 2:**
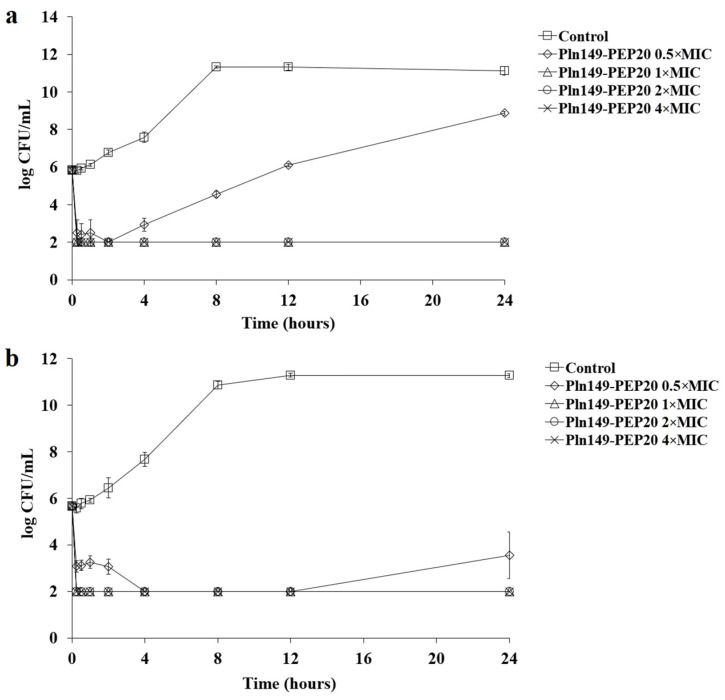
Pln149-PEP20 time-kill against (**a**) *A. baumannii* ATCC 19606 (MIC = 32 mg/L) and (**b**) *A. baumannii* ACI50 (MIC = 64 mg/L).

To better characterize the antimicrobial activity against Gram-positive and Gram-negative bacteria, the time-kill of AMP against *S. aureus* and *A. baumannii* at different concentrations relative to the MIC was assessed (Figure 1 and Figure 2).

The peptide had a quick bactericidal action, with a reduction of >3 logs of the inoculum in the first hour for all inhibitory concentrations. This reduction is similar to that observed for polymyxin B [16]. As expected, all samples treated with Pln149-PEP20 had statistically different total biomass compared to the untreated control. While the subinhibitory concentration obtained *p* < 0.05 compared with the biomass of the untreated control, the inhibitory concentrations were *p* < 0.001. Furthermore, in the reference strains used (*S. aureus* ATCC 25923 and *A. baumannii* ATCC 19606), the biomass obtained from the 0.5 × MIC curve can be considered different from the 1 × MIC biomass with *p* < 0.05. However, in the clinical strains (*S. aureus* SA43 and *A. baumannii* ACI50), the effect of the 0.5 × MIC concentration was more pronounced, and the biomass of 0.5 × MIC could not be considered different from that of 1 × MIC.

A Post-Antibiotic effect (PAE) assay was performed to determine the effect of Pln149-PEP20 on bacterial growth after treatment (Table 2).

Both subinhibitory and inhibitory concentrations presented equivalent PAE within the same strain (no statistical difference). Although a significant difference was observed in the 0.5 × MIC between *A. baumannii* ATCC 19606 and ACI50 (*p* = 0.045), the PAE was considered equivalent for both strains at 1 × MIC.

### 2.3. Cytotoxicity by MTS Assay

The selectivity index was calculated to determine how selective the molecule is—meaning, how the peptide’s activity affects human cells. Although the selectivity index was not calculated for HepG2, these cells were included in the study to verify activity against cancer cells. Pln149-PEP20 was tested, since Pln149 shares some similarities with PlnA, and this peptide has anticancer activity reported [19]. The anticancer activity of PlnA is mainly related to membrane activity that leads to apoptosis or necrosis—if Pln149-PEP20 interacts with zwitterionic lipids, then anticancer activity would be a possibility.

The concentration that reduced cell viability by 50% (CC_50_) obtained for Pln149-PEP20 was 67.73 ± 1.20 mg/L for THP-1 (differentiated human macrophages), 58.58 ± 1.95 mg/L for HFF-1 (human fibroblasts), and 125.15 ± 1.60 mg/L for HepG2 (human liver carcinoma). The selectivity index (SI) obtained from the data is shown in Table 3.

### 2.4. Synergism Assays

Pln149-PEP20 did not exhibit any synergism or antagonism when tested against *S. aureus* (ATCC 25923). Only indifferent interactions were observed, which allowed us to infer that Pln149-PEP20 does not compete directly with the targets of any of these antibiotics. For *A. baumannii* ATCC 19606, Pln149-PEP20 showed synergism with ciprofloxacin, polymyxin B, ampicillin, and vancomycin. The fractional inhibitory concentration (FIC) values are presented in Table 4. No antagonism was observed.

Synergism with vancomycin was investigated further. Although this antibiotic is used primarily for treating Gram-positive infections, investigating this synergism may help clarify the mechanism of action of Pln149-PEP20. In addition, potentiating molecules that can increase the spectrum of antibiotics already well established in clinical use is an important strategy to combat antimicrobial resistance.

Synergism with vancomycin was observed in other species (Table 5), such as *E. coli* and *K. pneumoniae*, except for polymyxin B-resistant *K. pneumoniae* AMKP4 and AMKP10.

**Figure 3:**
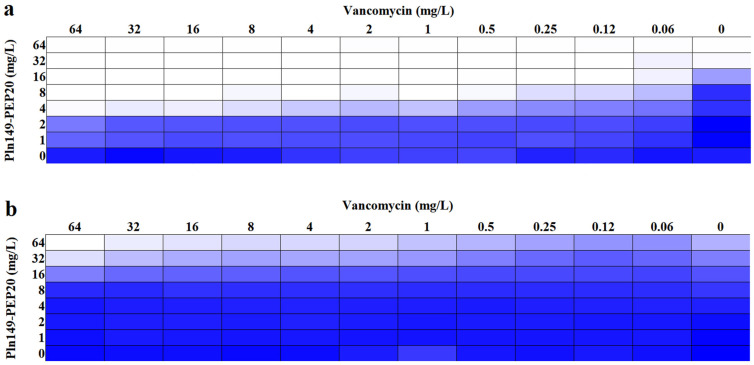
Heat map of synergism between Pln149-PEP20 and vancomycin (**a**) without and (**b**) with the addition of Mg^+2^ for *A. baumannii* ATCC 19606. The blue color represents the average absorbance of bacterial growth of three biological replicates.

A synergism assay with vancomycin was performed in the presence of excess magnesium to assess whether these ions directly influence the action of the peptide on the outer membrane. This assay is represented as a heat map in Figure 3. The presence of exogenous Mg^+2^ prevented synergism with vancomycin and prevented the action of Pln149-PEP20 itself. The presence of this cation in excess indeed prevents Pln149-PEP20 from establishing interactions that are fundamental for its activity. Bacterial membranes have divalent cations, such as Mg^+2^ and Ca^+2^, that interact and stabilize the membrane on its outer facet. Cationic AMPs, such as Pln149-PEP20, compete with these ions for electrostatic interactions, displacing them and establishing initial interactions with negative components [21].

### 2.5. Membrane Depolarization Assay

**Figure 4:**
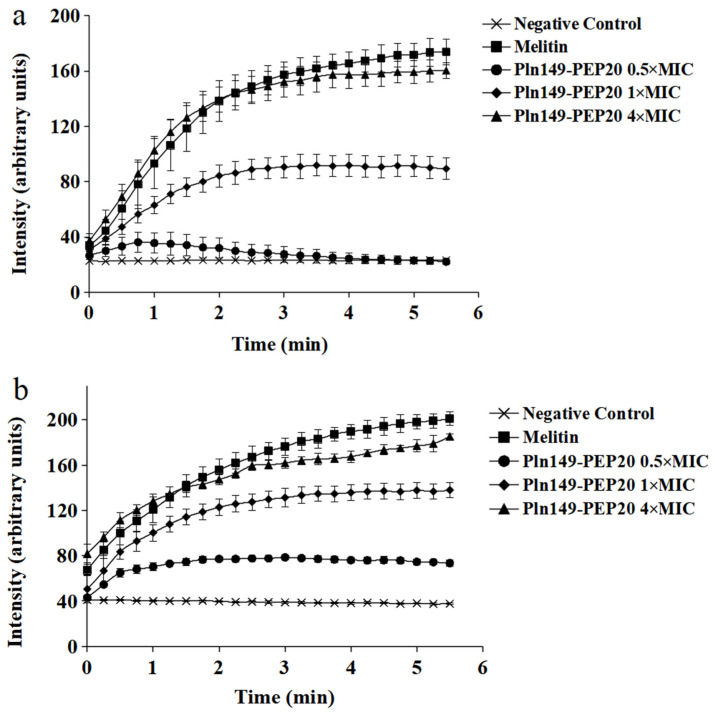
Pln149-PEP20 cytoplasmic membrane depolarization for (**a**) *S. aureus* ATCC 25923 (MIC = 8 mg/L). (**b**) *A. baumannii* ATCC 19606 (MIC = 32 mg/L).

The ability of Pln149-PEP20 to depolarize the membrane was evaluated using DISC3(5) dye. The intensity of the signal, which is proportional to the depolarization of the cytoplasmic membrane, is presented in Figure 4. The peptide was able to depolarize both Gram-positive and Gram-negative bacteria in less than 5 min. The 4 × MIC concentration of the peptide presented an area under the curve like that of melittin (*p* > 0.05) for both species, indicating that complete depolarization occurred under these conditions. In contrast, the 0.5 × MIC concentration in *S. aureus* ATCC 25923 did not present a statistically significant difference compared to the negative control area (*p* > 0.05).

### 2.6. Circular Dichroism

**Figure 5:**
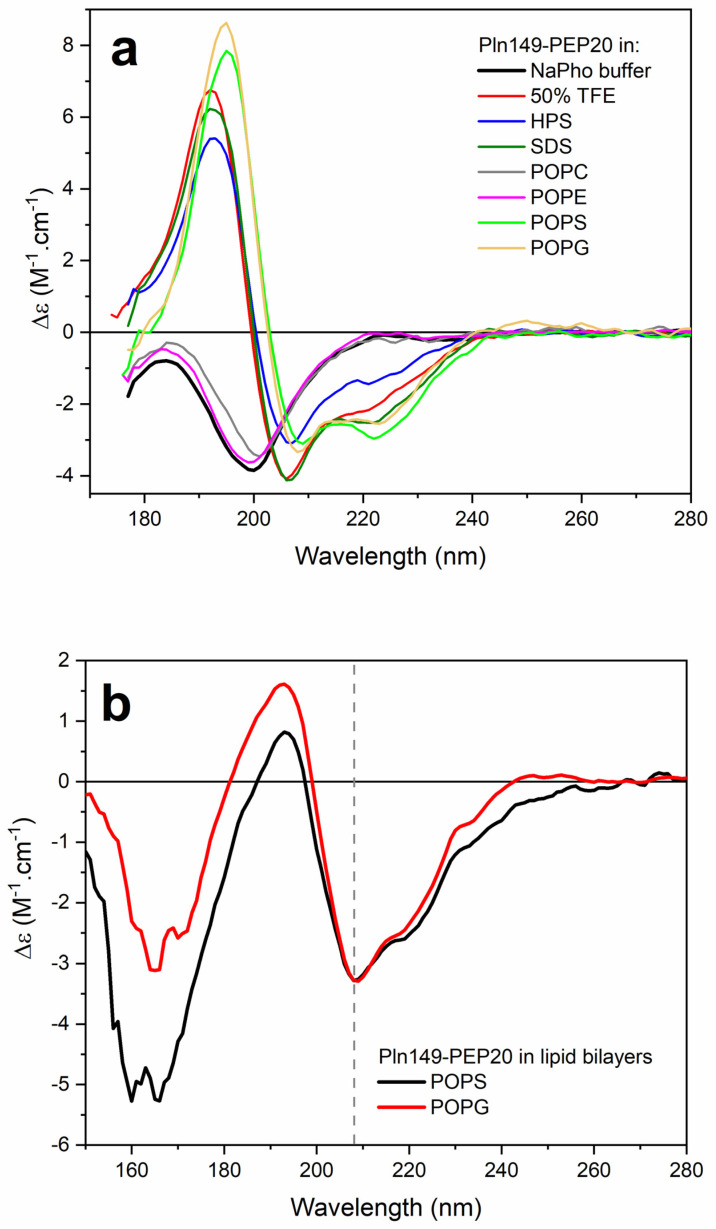
(**a**) SRCD spectra of Pln149-PEP20 in 10 mM sodium phosphate buffer pH 7 (black), 50% TFE (red), in the presence of the surfactants sodium dodecyl sulfate (SDS, olive) or N−hexadecyl−N−N’ dimethyl−3−ammonia−1−propane−sulfonate (HPS, blue), or vesicles of POPC (gray), POPE (magenta), POPS (green), and POPG (orange), at a 1/50 peptide-to-lipid molar ratio. (**b**) o-SRCD spectra of PEP20 in lipid bilayers of POPS (black) and POPG (red).

**Figure A1:**
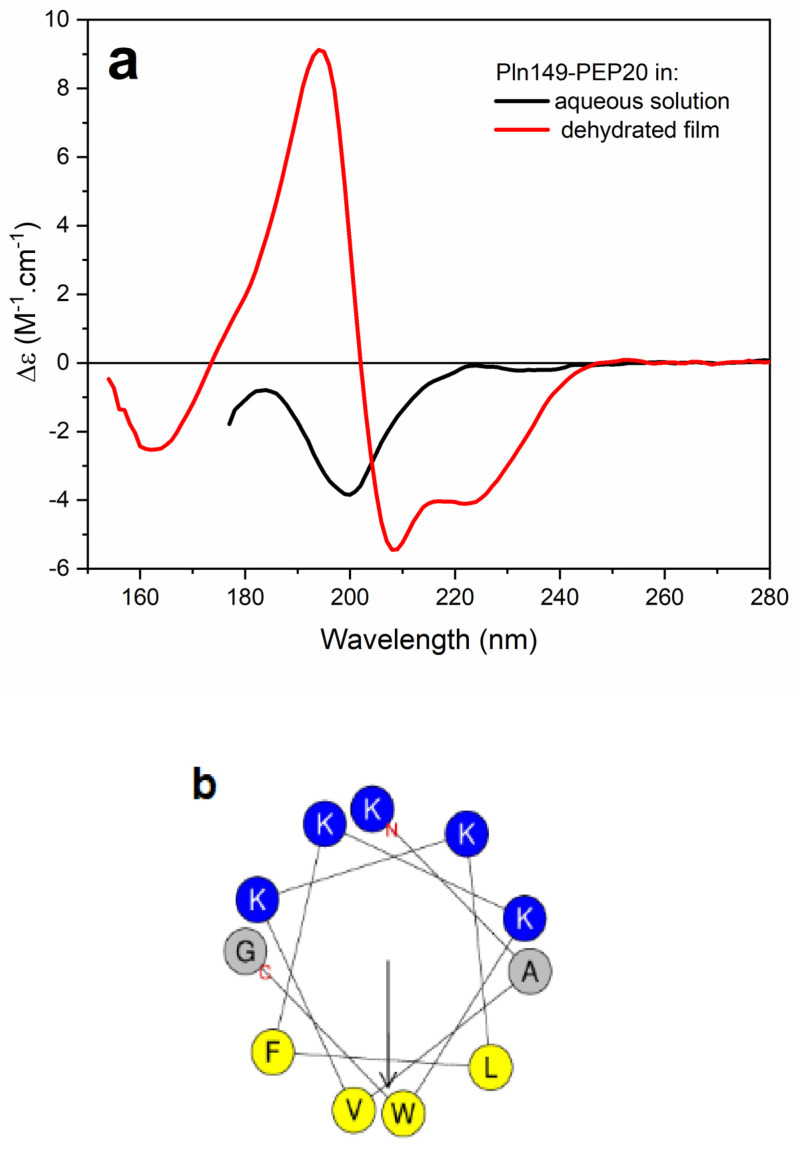
(**a**) SRCD spectra of Pln149-PEP20 in sodium phosphate buffer pH7 (black) and in the semidehydrated film on the surface of a quartz−Suprasil plate (red). (**b**) Helical wheel projection of the Pln149-PEP20 sequence. Nonpolar residues are labelled in yellow, positively charged residues are in blue.

To understand how the new peptide interacts with membranes, Pln149-PEP20 was characterized biophysically. The synchrotron radiation circular dichroism (SRCD) spectroscopy spectrum of Pln149-PEP20 was obtained in an aqueous solution (Figure 5a). Pln149-PEP20 in aqueous solution showed peaks attributed to the disordered peptide content [22]. However, a disordered-to-helix transition in the Pln149-PEP20 secondary structure was observed in the presence of 50% trifluoroethanol (TFE) or when the peptide formed a partially dehydrated film (Appendix B, Figure A1) due to the favored intramolecular hydrogen bonds [10]. The induced alpha helix in the Pln149-PEP20 structure shows off its amphipathic character (Appendix B, Figure A1) to interact at lipid interfaces.

Distinct model membranes were employed to investigate the binding of peptide Pln149-PEP20 to lipid systems in aqueous solutions and oriented lipid bilayers. The peptide assumed a stable α-helical structure in the presence of negatively charged or zwitterionic surfactants. In an aqueous solution, no conformational changes in Pln149-PEP20 were observed in the presence of zwitterionic large unilamellar vesicles (LUVs) of 1-palmitoyl-2-oleoyl-phosphocholine (POPC) and 1-palmitoyl-2-oleoyl-phosphoethanolamine (POPE) (Figure 5a). However, the two minima at 222 nm and 208 nm and a positive maximum at 192 nm were observed in the peptide SRCD spectra in the presence of the vesicles of 1-palmitoyl-2-oleoyl-phosphoglycerol (POPG) and 1-palmitoyl-2-oleoyl-phospho-L-serine (POPS). This observation revealed the partition of the peptide to the negatively charged vesicles.

o-SRCD spectroscopy was used to characterize the alignment of the α-helix induced in Pln149-PEP20 in the macroscopically oriented lipid bilayers of POPG and POPS. The o-SRCD spectra of the peptide in both negatively charged bilayers (Figure 5b) exhibited two minima at 208 nm and 220 nm, with a more pronounced negative value at 208 nm. This spectral line shape is typical of peptides assuming a surface-bound state, S-state [23], and aligned parallel to the plane of the lipid bilayers.

### 2.7. Transmission Electron Microscopy (TEM)

**Figure 6:**
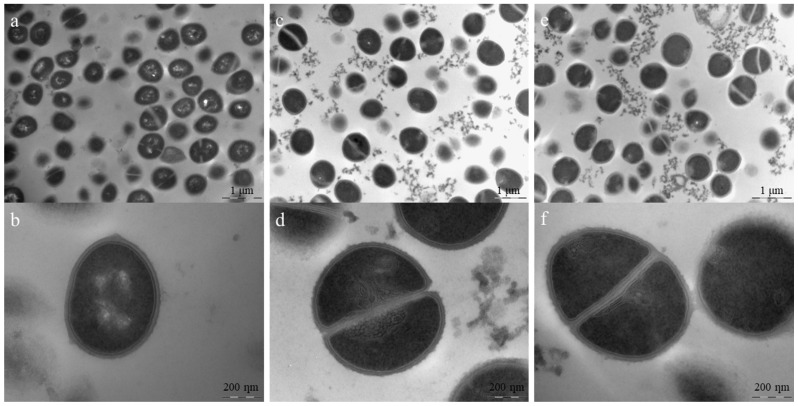
TEM for *S. aureus* ATCC 25923 treated with Pln149-PEP20 (MIC = 8 mg/L). (**a**) non-treated cells. Scale bar = 1 µm (**b**) non-treated cell in detail, with intact cell wall and membrane. Scale bar = 200 nm (**c**) 1 × MIC treated cells, showing cell debris and deficient cells. Scale bar = 1 µm (**d**) 1 × MIC treated cells in detail, showing cell debris, cell with defective division septum, and intralamellar structures. Scale bar = 200 nm (**e**) 4 × MIC treated cells, showing cell debris and deficient cells. Scale bar = 1 µm (**f**) 4 × MIC treated cells in detail, showing cell with defective division septum, intralamellar structures, and diffuse membrane. Scale bar = 200 nm.

TEM helped us better understand the mode of action of the AMP in bacterial membranes. In S. aureus, membrane damage, intralamellar structures resembling mesosomes, and errors in the division septum were observed in most cells (Figure 6). Division septum errors also occurred for the positive control, but at a frequency of 21 ± 10%, whereas cells treated with 1 × MIC and 4 × MIC presented 61 ± 20% and 54 ± 12%, respectively. This difference in frequency is significantly different from the positive control (*p* < 10^−4^). Mesosomes can occur in response to membrane damage (due to phospholipid reorganization) and cell wall damage (similar patterns seen in vancomycin and tetracycline studies [24]. Therefore, this peptide may also affect cell wall biosynthesis. As the machinery responsible for cell wall biosynthesis is in the division septum [25], it is common to observe these mesosomes near this structure.

**Figure 7:**
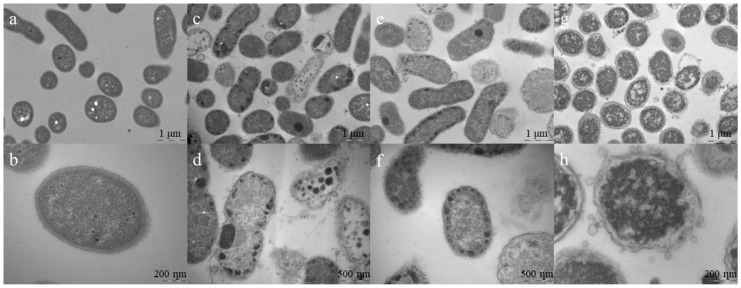
TEM for *A. baumannii* ATCC 19606 treated with Pln149-PEP20 (MIC = 32 mg/L). (**a**) non-treated cells. Scale bar = 1 µm (**b**) non-treated cell in detail, with homogeneous intracellular content. Scale bar = 200 nm (**c**) 0.5 × MIC treated cells, showing some cell debris and condensation of cytoplasmic content. Scale bar = 1 µm (**d**) 0.5 × MIC treated cells, showing breakage of cell wall and membrane, besides condensation of cytoplasmic content in the peripheral area of cells. Scale bar = 500 nm (**e**) 1 × MIC treated cells, showing peripheral cytoplasmic condensation. Scale bar = 1 µm (**f**) 1 × MIC treated cells, showing peripheral cytoplasmic condensation and diffuse membranes. Scale bar = 500 nm (**g**) 4 × MIC treated cells, showing a heterogenous intracellular content distribution and bleb formation, typical of membrane disturbing treatments. Scale bar = 1 µm (**h**) 4 × MIC treated cell, with completely disturbed cell wall and membranes, surrounded by blebs. Scale bar = 200 nm.

In *A. baumannii*-treated cells (Figure 7), damage to both the outer and inner membranes was evident at higher peptide concentrations, with wrinkled surfaces and bleb formation. In lower concentrations, cytoplasmic material condensation can be observed in specific positions in the cell. These positions coincide with those of specific proteins, such as PlsX, FloT, and MurG [26]. These proteins are related to the cell membrane or cell wall, suggesting Pln149-PEP20 targets. Although it is impossible to determine from the TEM experiment in which protein the interaction occurs, the carpet mode of action explains the rapid membrane depolarization. The binding of Pln149-PEP20 to MurG would explain the other forms of damage observed. Such damage was usually associated with the cell wall. Thus, MurG is a strong candidate for investigation.

### 2.8. In Vitro Directed Evolution and Genome Analysis

**Figure 8:**
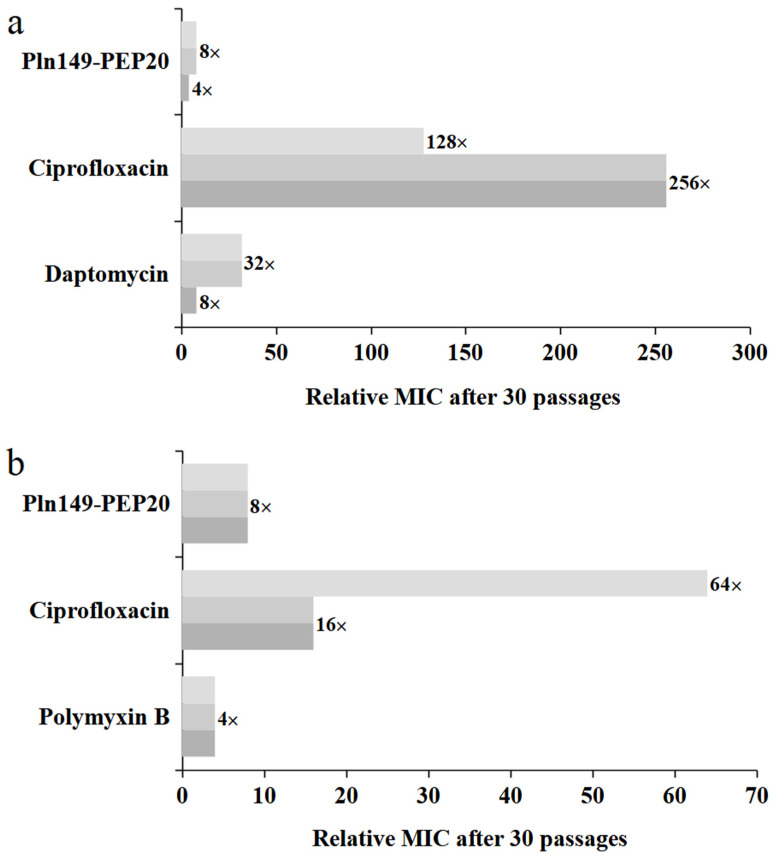
Relative MIC increase for each 30-day exposure, including exposure with Pln149-PEP20, each replicate represented in a shade of gray. (**a**) *S. aureus* ATCC 25923 (**b**) *A. baumannii* ATCC 19606.

The propensity of the peptide to select resistant isolates was evaluated by increasing the subinhibitory concentrations of the antimicrobial agent over 30 days. The increase in MIC is shown in Figure 8 for both Gram-positive and Gram-negative bacteria. The peptides exhibited a moderate propensity to select resistants. The MIC increments observed were comparable to those of daptomycin and polymyxin B, which are last-resort antibiotics with peptide composition and action on the cell wall and membrane, as suggested for Pln149-PEP20.

The initial and final strains with reduced susceptibility to Pln149-PEP20 were sequenced to determine whether mutations related to the mode of action occurred. All mutations obtained for *S. aureus* and *A. baumannii* are listed in Table 6 and Table 7, respectively.

There was no mutation in common between all biological replicas in *S. aureus*, but the DUF1672 domain protein appeared in two of the three final strains, which makes it a strong candidate for future investigation as a target for Pln149-PEP20. All mutations in *A. baumannii* occurred in the gene encoding an Ig-like domain-containing protein, which implies this has a critical role in the peptide’s interaction or action.

## 3. Discussion

Some Plantaricin 149 analogs previously explored showed antimicrobial activity, but this was not investigated thoroughly. When such activity was observed, it occurred at high concentrations. An Fmoc protecting group at the N-terminus of the peptide is noteworthy and seems to have increased the activity of this bacteriocin [9]. This group is used in organic synthesis to protect amines, and has hydrophobic characteristics. Thus, the first peptide to be tested was Fmoc-Pln149, the original sequence of this bacteriocin with the Fmoc-protecting group. This peptide was active against all the strains tested, but with hemolytic activity occurring at much lower concentrations than the action against pathogenic bacteria. Fmoc-Pln149(6-22) lacked the N-terminal portion of the peptide, based on studies showing that this region does not contribute to antimicrobial activity in PlnA [8]. Fmoc-Pln149(6-22) peptide showed activity against all tested strains. Despite acting at higher concentrations than those of Fmoc-Pln149, its hemolytic activity was attenuated. Therefore, the Fmoc group is not solely responsible for the hemolytic activity, as the amino-acid sequence also influenced this. The peptide Pln149-PEP20 proposed in this paper was based on Fmoc-Pln149(6-22) but used alpha-helix projection and altered the amino acids to obtain a well-defined amphipathic secondary structure. This peptide showed a significant improvement compared with previously published peptides because it is not only bactericidal at lower concentrations, but it also significantly reduces the hemolytic rate. Therefore, Pln149-PEP20 was more advantageous than the parent molecule, and its antimicrobial activity was characterized in this study.

The investigation of Pln149-PEP20 allowed us to determine the peptide’s antimicrobial activity characteristics. The PAE results indicated that, even after the serum concentration decreased, the peptide continued to prevent bacterial growth [27]. Pln149-PEP20 seemed to maintain an action on bacterial growth to the same degree as polymyxin B. The similarity of PAE and time-kill results between the peptide and polymyxin B suggests that Pln149-PEP20 could have similar pharmacodynamic properties to those reported for polymyxin B. Both polymyxin B and Pln149-PEP20 can be classified as concentration-dependent, meaning they are more effective if they reach a concentration higher than the MIC. However, the time at which the concentration is maintained is less important than the concentration level [16].

As AMPs are reported to be versatile in their action spectrum, we also investigated the activity of the proposed molecule against cancer cells. Although no activity against HepG2 was observed, assays that include other cells must be performed to exclude anticancer action. In some cancer cells, the flip-flop phenomenon occurs, in which there is an exchange between phospholipids from the inner and outer leaflets of the eukaryotic cytoplasmic membrane. Consequently, changes occur in the phospholipid constitution, membrane fluidity, and surface charge. These changes can attract AMPs, as observed in the bacterial membrane [28]. In addition, other plantaricins also had activity against cancer cells, encouraging investigation for the newly designed peptide [19].

The assay against eukaryotic cells also showed that Pln149-PEP20 selectivity was lower than the hemolysis assay initially suggested. Ideally, an IS ≥ 10 is expected to be considered a biologically effective antibiotic [29]. The hydrophobic surface of this molecule may interact with both cell membranes [10]. Beyond that, the outer membrane could be an obstacle to the peptide, explaining the higher MICs against Gram-negative bacteria.

The synergism of commercial antibiotics was assessed to obtain a better selectivity index for these Gram-negative bacteria. The concomitant therapeutic use of antibiotics has been an alternative to overcome the problem of antimicrobial resistance [3]. Synergism with vancomycin was further explored because, besides having the lowest FIC index, this may indicate that Pln149-PEP20 acts on the outer membrane. Vancomycin is a glycopeptide that inhibits cell wall synthesis by binding to the D-ala-D-ala portion of the cell wall precursor, preventing the action of transpeptidases and peptidoglycan polymerases and thus inhibiting the cross-linking of peptidoglycans that make up the cell wall [30]. Vancomycin could have a broad spectrum of activity [31]; however, the Gram-negative outer membrane hinders the antibiotic from permeating the periplasmic space. Therefore, the synergism observed was likely due to the outer membrane destabilization caused by Pln149-PEP20, which allowed vancomycin to act on the cell wall.

Of all strains tested, only three did not show Pln149-PEP20 synergism with vancomycin. The lack of synergism in *P. aeruginosa* can be explained by the low permeability of its outer membrane [32]. For the other two strains, both are polymyxin-resistant, which could have led to lower Pln149-PEP20 activity. The polymyxin resistance mechanism in these strains is due to an insertion element in the *mgrB* gene. Therefore, these strains have an increased surface charge due to the addition of 4-amino-4-deoxy-L-arabinose to lipid A. Despite also being resistant to polymyxins, ACI50 harbors mutations in the *pmrCAB* system [20], resulting in a lower charge difference than that caused by the insertion element disruption of the *mgrB* gene in *K. pneumoniae* isolates [18,33]. That could explain why *A. baumannii* ACI50 has a lower FIC index than the other polimixin-resistant strains.

After characterizing the peptide’s action, we tried to elucidate how it acts. The investigation of membrane depolarization allowed for a better understanding of peptide interactions. Although the 0.5 × MIC concentration did not cause relevant depolarization in *S. aureus*, the time-kill results showed that this concentration could reduce the bacterial population. Therefore, in the absence of significant depolarization, one can infer that Pln149-PEP20 has other undiscovered mechanisms of action. Depolarization occurred in increments, likely because of the peptide mode of interaction with the membrane.

The SCRD data was a way to evaluate in vitro the interaction of this new peptide with membranes. The binding of Pln149-PEP20 was also guided by electrostatic interactions with the lipid surface, especially in highly packed lipid systems. These findings agree with the higher activity of the peptide against Gram-positive bacteria.

Recent data show that Plantaricin 149 acts via the carpet mode of action [10], in which the peptides interact with the membrane surface (usually with the hydrophobic part facing the membrane and the hydrophilic part facing the solvent phase). The o-SCRD for Pln149-PEP20 revealed that the helix was in contact with the lipid headgroups during the entire membrane permeation process and was not inserted across the membrane hydrophobic core, compatible with the carpet mode of action as well. Pln149-PEP20 binding might promote peptide accumulation at the lipid surface until it reaches a threshold concentration at which the membrane is efficiently disturbed, as described by the carpet-like model. Thus, depolarization increments can be explained by the SCRD data—they represent the threshold concentrations that need to be achieved in the membrane.

In vitro selection showed not only the peptide’s low propensity to cause resistance compared to conventional antibiotics. The assay also helped clarify the mechanism of action of Pln149-PEP20 by allowing us to investigate what kind of alterations can lead to a lower sensitivity to the molecule.

It is unclear which of the mutations obtained for the in vitro-selected strains is essential for Pln149-PEP20 sensitivity reduction in *S. aureus*. Although CflA has a highly negative region due to aspartate charges, its contribution to the cell surface charge has never been reported. CflA and FlbPA are essential virulence proteins of *S. aureus* that adhere to human body proteins, such as elastin, fibronectin, and fibrinogen, during the infection process [34]. The latter commonly occurs in both CflA and FlbPA. A mutation in FlbPA occurs in the A subunit, which binds to fibrinogen. No evidence exists for the interaction of Pln149-PEP20 with fibrinogen binding sites, mainly because this molecule is highly anionic at physiological pH, unlike AMPs. Therefore, it is likely that these mutations were selected as a form of regulation because Pln149-PEP20 susceptibility decreases. Studies have already reported that MRSA has a lower expression of virulence factors when resistant to daptomycin than when sensitive to the same antibiotic. This lower expression guarantees a long and persistent infection versus an acute infection in the short term [35,36].

The DUF1672 domain-containing protein has an important function related to Pln149-PEP20, given that a mutation occurs in two different biological replicas of the assay. Recent studies indicate that this family is a constituent of the lipoproteome of *S. aureus* [37], and that this domain can encode lipoproteins that are added to the outer facet of the lipid bilayer. Although much remains to be characterized about these lipoproteins, they are associated with cell wall synthesis, electron transport, nutrients, and even surface stress response [38]. This finding reinforces previous results related to Pln149-PEP20’s mechanism of action.

The bifunctional (p)ppGpp/guanosine-3′-5′-bis(diphosphate)-3′-pyrophosphohydrolase is intrinsically related to the stringent response. They are usually synthesized in response to nutrient limitation, rRNA degradation, enzymatic activation, and replication [39]. There is a relationship between the expression of alarmones and starvation reactions due to a lack of amino acids or as a response to cell wall stresses. The transcription of the alarmones is related to tolerance to antibiotics that act on the cell wall, such as vancomycin and ampicillin. These antibiotics anticipate the response to oxidative stress, allowing the survival of the cell [40,41].

The role of the FAD-containing oxidoreductase mutation in in vitro selection remains unclear. Several FAD oxidoreductases are expressed by *S. aureus*. These oxidoreductases protect the cell against massive oxidative damage by degrading reactive oxygen species [42,43]. The mutation in this protein can be regarded as conservative because of the exchange of a hydrophilic amino acid (serine) for one that is also hydrophilic (threonine). Only a methyl group differentiates these two amino acids. It is, therefore, unclear whether this mutation is directly related to reduced susceptibility to Pln149-PEP20.

The major facilitator superfamily is one of the mutations that stand out in directed evolution. Members of this family are essential for transporting various molecules across the membranes. This transport depends on several conformational changes caused by the claw mechanism. Its substrates vary from nutrients, metabolites, and signaling molecules to toxins and drugs [44]. The mutation in this protein may have caused a loss of function, as it caused a change in the reading frame. Thus, it is assumed that the strain with mutated transporters gained an evolutionary advantage by lowering the importation of Pln149-PEP20 into the cell. This leads to the critical conclusion that Pln149-PEP20 is likely to have an intracellular target in addition to the membrane damage already observed.

For *A. baumannii*, all mutations occurred in the same target. The Ig-like domain-containing protein in which mutations occurred contains conserved domains of Ca^+2^-stabilized adhesin repeat [45,46]. Although many proteins contain this domain, these mutations can be related to the presence of stabilizing ions in the outer facet of the membrane, where calcium plays a fundamental role. Our previous assays suggested that more stabilizing ions may affect Pln149-PEP20 activity.

The focus of this paper was the extensive characterization of antimicrobial action, but the research would benefit from further assessment. For example, bacterial cytological profiling [26] could aid in investigating the mechanism of action and shed light on some hitherto unexplained results, such as whether Pln149-PEP20 acts intracellularly, or what effect on Gram-negative cells causes condensation of cytoplasmic contents.

The assessment of cytotoxicity in the research is still limited. The Fmoc group has been used based on previous results but is associated with high cytotoxicity [47]. It could be beneficial to investigate a group with similar characteristics to replace the N-terminal portion of the peptide. Although the combination therapy strategy was the chosen approach in this research, many others can still be applied in the future, such as conjugation strategies, to not only combat the adverse effects of the peptide, but also deliver active molecules with optimal biocompatibility [48].

Thus, the present study should be a starting point for future optimizations until in vivo assays and possible applications.

## 4. Materials and Methods

### 4.1. Peptide Synthesis

Using 9-fluorenylmethyloxycarbonyl (Fmoc) chemistry, Pln149-PEP20 was manually synthesized using solid-phase peptide synthesis, as described by [49]. The synthesis involves sequential coupling and deprotection reactions. Initially, the Rink Amide resin was solvated in N,N-dimethylformamide (DMF) and dichloromethane (DCM). Deprotection was performed using 20% 4-methyl-piperidine in DMF. The couplings were then carried out, solubilizing each Fmoc-amino acid, the coupling activators N-Hydroxybenzotriazole (HOBT), and diisopropylcarbodiimide (DIC) in DMF and DCM (2 mol of amino acid: 1 mol of resin). The coupling and deprotection steps were repeated until all the amino acids had been added to the peptidyl-resin. The ninhydrin test was performed after each coupling/deprotection step to verify its efficiency (Kaiser et al., 1970). After coupling the last amino acid residue, the peptide from the resin was cleaved for 2 h using a solution of 95% trifluoroacetic acid (TFA), 2.5% triisopropylsilane, and 2.5% water. The crude peptide was precipitated into cold diethyl ether, extracted with 0.045% TFA (*v/v*) in water, and lyophilized. The purification of crude Pln149-PEP20 was performed in semi-preparative mode, using a Shimadzu HPLC System in a C18 reverse-phase column (Phenomenex 2.1 × 25 cm). The column had previously been equilibrated with 0.1% TFA in water and eluted, using a linear gradient from 0 to 70% acetonitrile 90% in water (with 0.1%TFA) and a flow rate of 1 mL/min. The absorbance at 220 nm and 280 nm was then monitored. Peptide identity was further confirmed by electrospray mass spectrometry using an ESImicroOTOF-Q instrument (Bruker Daltonics). Peptides were solubilized in formic acid 0.5% (flow of 180 µL/h and capillary voltage of 4500 V) and analyzed in the 50–3000 *m*/*z* range.

### 4.2. Bacterial Strains

Species that represent the main pathogenic organisms that threaten public health were selected for this study. ATCC strains were used because they are well characterized and are used as a reference for determining several antibiotics’ minimum inhibitory concentrations (MIC). For most of the assays, *S. aureus* ATCC 25923, *A. baumannii* ATCC 19606, and two clinical strains previously characterized by our research group, MRSA *S. aureus* SA43 [50], and the extensively drug-resistant (including polymyxin B-resistant) *A. baumannii* ACI50 [20] were used. More strains characterized by Dabul et al. (2018), Kuroda et al. (2001), Dabul et al. (2019), de Mello et al. (2016), Mello et al. (2020), Souza et al. (2019), Carrasco et al. (2021), and Galetti et al. (2016) [17,18,20,51,52,53,54,55,56] were included in Section 2.3 and are described in detail in Table A1 and Table A2.

### 4.3. Quantitative Antimicrobial Susceptibility

The MIC of Pln149-PEP20 was determined using the broth microdilution method described by the Clinical and Laboratory Standards Institute [57]. The peptides were diluted in cation-adjusted Mueller-Hinton (CAMH) broth (BD, East Rutherford, NJ, USA), ranging from 512 mg/L to 0.06 mg/L. The bacterial inoculum was added to the peptide, resulting in a 5 × 10^5^ CFU/mL concentration in each well. The descriptions of the bacteria used in this study are provided in Table A1 and Table A2. The MIC was determined as the lowest concentration that inhibited visible microbial growth after incubation at 35 °C for 24 h. All assays were performed in triplicate, and polystyrene U-bottom microplates with minimal protein binding were used. Bacterial growth without peptides was used as a negative control. Daptomycin and polymyxin B were used as positive controls to compare the known MIC of ATCC strains described in the CLSI [58].

A total of 100 μL from each well without growth in the MIC assay was subcultured on CAMH agar plates, which were incubated at 35 °C for 24 h to determine the minimum bactericidal concentration (MBC). MBC was the lowest peptide concentration, with no visible growth on the plate. For initial comparison, the MIC of Fmoc-Pln149 and Fmoc-Pln149(6-22) were also determined for the ATCC strains.

### 4.4. Hemolytic Activity

The hemolytic activity of Pln149-PEP20 was determined according to the protocol described by Castro et al. [58]. This study was approved by the Ethics Committee of the Federal University of São Carlos (CAAE 52231421.7.0000.5504). Blood from human volunteers who had not been treated with medication was collected in tubes with ethylenediaminetetraacetic acid (EDTA) and washed three times with phosphate-buffered saline (PBS). The precipitated cells were resuspended in 1% PBS. Erythrocytes were incubated with the peptide at concentrations ranging from 512 mg/L to 0.06 mg/L for 1 h at 37 °C. Triton X-100 1% was used as a positive control for hemolysis. After incubation, the microplate was centrifuged. The hemolytic rate was determined by measuring the absorbance of the supernatant at 405 nm. The percentage of hemolysis was calculated using the following equation:

%Hemolysis = 100 × [(Sample-blank)/(Triton-blank)],(1)

The HC_50_ value was defined as the peptide concentration that produced 50% hemolysis. The assays were performed in technical and biological triplicate. HC50 was determined by logarithmic regression using GraphPad Prism software.

### 4.5. Time-Kill Assay

The experiments were performed as recommended by the CLSI [56]. The activity of Pln149-PEP20 over time was determined against two species associated with a high burden of MDR infections: S. aureus ATCC 25923 and SA43 strains [50] and *A. baumannii* ATCC 19606 and ACI50 strains [20]. Concentrations of 0.5×, 1×, 2×, and 4× the MIC of the peptide or antibiotics were used against an inoculum of 6 × 10^5^ CFU/mL. Aliquots (20 µL) were collected at 0, 15, and 30 min, and 1, 2, 4, 8, 12, and 24 h. These were serially diluted (1:10) in 0.85% sterilized saline, which was then cultivated using the micro-drop technique (6 drops of 15 µL each) on brain and heart infusion (BHI) agar and incubated at 37 °C for 24 h. The colonies were counted later. Bacterial growth without the peptide was a negative control, and daptomycin and polymyxin B were used as positive controls. The experiment was performed in duplicate, using biological and technical sextuplets. The detection limit of this experiment was 10^2^ CFU/mL. After plotting the CFU/mL × time of the killing kinetic assay, the area under the curve was calculated using MATLAB^®^ R2021b for comparison. The area under the curve was proportional to the total biomass present in the assay. Biomass at different concentrations was compared using analysis of variance (ANOVA).

### 4.6. Post-Antibiotic Effect Assay

Post-antibiotic effects were evaluated as described by Saravolatz et al. [27], using the same bacteria as in the time-kill assay. An inoculum of 100 µL of 6 × 10^7^ CFU/mL adjusted bacteria was added to 10 mL of MHCA that contained 0.5× and 1× the MIC of the peptide. After homogenization, the tubes were incubated at 37 °C for 10 min and centrifuged at 3000× *g* for 10 min at room temperature. The supernatants were discarded, and the bacteria were resuspended in 10 mL of fresh MHCA at 37 °C. A 20 µL inoculum was taken and serially diluted in 0.85% saline every hour. Dilutions were cultivated using the micro-drop technique (six drops of 15 µL each) on BHI agar and incubated at 37 °C for 24 h. The colonies were counted later. Bacterial growth without peptides was used as the negative control. Daptomycin and polymyxin B were the positive controls. The post-antibiotic effect (PAE) is defined as:

PAE = T − C.(2)

In this equation, T is the time for the treated sample to increase by 1 log, while C is the same, but for the growth control. Assays were performed in biological duplicates and in technical sextuplets. The concentrations used in this assay and the different strains treated were compared using ANOVA, with a threshold of *p* < 0.05.

### 4.7. Cytotoxicity by MTS Assay

Cytotoxicity evaluation using [3-(4,5-dimethylthiazol-2-yl)-5-(3-carboxymethoxyphenyl)-2-(4-sulfonyl)-2H-tetrazolium] (MTS) was performed using THP-1 (differentiated human macrophages), HFF-1 (human fibroblasts), and HepG2 (human liver carcinoma) cells. Cells were grown in 96-well plates at 37 °C for 24 h. The peptide was added, ranging from 0.06 mg/L to 512 mg/L, and incubated for 24 h at 37 °C. MTS was added, and the cells were incubated for 4 h. Absorbance was measured at 490 nm using a SpectraMax 384 spectrophotometer (Sunnyvale, CA, USA; Valli et al., 2022). Data were analyzed using GraphPad Prism 8.0 for the calculation of CC_50_ (concentration that reduced cell viability by 50%). The percentage of nonviable cells was determined and compared to that of the negative control wells (100% growth). All assays were performed in triplicate, and doxorubicin (Sigma-Aldrich, St. Louis, MO, USA) was used as a positive control. The selectivity index was calculated by the ratio between the CC_50_ and MIC [59]. A measure of MIC_50_ was used to determine the ratio, which is the minimum concentration required to inhibit at least 50% of all bacterial strains tested within a given group.

### 4.8. Synergism Assay

The investigation of synergism was performed through checkerboard analysis, using the same method described in Section 4.3 but with the dilution of Pln149-PEP20 (compound A) horizontally and antibiotics (compound B) vertically in the same microplate. Ciprofloxacin, tobramycin, vancomycin, ampicillin, and imipenem were tested, along with the peptides, against *S. aureus* ATCC 25923 and *A. baumannii* ATCC 19606. Daptomycin and polymyxin B were tested against the Gram-positive and Gram-negative strains, respectively. The fractional inhibitory concentration (FIC) was calculated using the formula:

(MIC^A^_combination_)/MIC^A^) + (MIC^B^_combination_)/MIC^B^) = FIC_A_ + FIC_B_ = FIC_index_. (3)

Synergism is a combination that results in an FIC < 0.5 [21]. In addition, CAMH supplemented with Mg^+2^ (magnesium chloride at a final concentration of 21 mM, as described by Li et al. [21]) was used to test if excess outer-membrane-stabilizing ions influence the vancomycin synergism mechanism. The reason for this addition is that the action of AMPs is typically associated with electrostatic interactions that displace stabilizing ions from the membrane. The presence of excess stabilizing ions, such as calcium and magnesium, can lead to competition for interactions and therefore undermine the action of AMP. Assays were performed in biological and technical triplicate.

### 4.9. Membrane Depolarization Assay

This assay was performed using 3,3′-dipropylthiadicarbocyanine iodide, DISC_3_(5), which allows for assessing cytoplasmic membrane depolarization. This assay was performed as previously described [60] for *S. aureus* ATCC 25923 and *A. baumannii* ATCC 19606. Single colonies were cultivated in MHCA with agitation at 37 °C until the mid-log phase (between 4 h and 6 h). The bacterial culture was centrifuged at 4000 rpm at room temperature for 10 min. The supernatant was discarded, and the cells were suspended in a respiration buffer (5 mM HEPES and 20 mM glucose, pH 7.4). The cells were again centrifuged and suspended in fresh buffer and then adjusted to OD_600_ = 0.05, using SpectraMax M5 (Molecular Devices). A stock solution of DISC_3_(5) was then added at a final concentration of 0.2 μM. For *A. baumannii*, 0.05 mM of EDTA was added. The cells were then incubated with DISC3(5) for 1 h at room temperature. After incubation, 200 μL was added to each well of a matte-black, flat-bottomed microplate. To each well, 2 µL of the peptide was added at a final concentration of 4×, 1×, or 0.5 × MIC. Melittin was used as a positive control at a final concentration of 10 mg/mL (100% depolarization, always compared to peak depolarization).

Assays were performed in biological and technical triplicate. The plate was read on a SpectraMax M5 (Molecular Devices) in fluorescence mode, with excitation at 622 nm and emission at 670 nm for 5 min. As in the time-kill method, the area under the curves of intensity × time was calculated using MATLAB R2021b for comparison. These were compared using ANOVA.

### 4.10. Synchrotron Radiation Circular Dichroism Spectroscopy (SRCD)

The SRCD spectra of Pln149-PEP20 were collected at the AU-CD beamline on the ASTRID2 synchrotron (University of Aarhus, Aarhus, Denmark) over the wavelength range of 280–170 nm, using a 1 nm interval in a 0.0101 cm pathlength quartz Suprasil cuvette (Hellma Scientific) at 25 °C. Three individual scans were recorded for each sample: Pln149-PEP20 (0.73 mM) incubated in 10 mM sodium phosphate buffer (pH 7), or the peptide in 50% trifluoroethanol (TFE), in the presence of membrane models composed of 20 mM sodium dodecyl sulfate (SDS) or N-hexadecyl-N-N’ dimethyl- 3-ammonia-1-propane-sulfonate (HPS), or in the presence of large unilamellar vesicles (LUVs) of 1-palmitoyl-2-oleoyl-phosphocholine (POPC), 1-palmitoyl-2-oleoyl-phosphoethanolamine (POPE), 1-palmitoyl-2-oleoyl-phospho-L-serine (POPS), and 1-palmitoyl-2-oleoyl-phosphoglycerol (POPG) at a 1/50 peptide-to-lipid molar ratio. Micelles and LUVs were prepared in sodium phosphate buffer (pH 7.0). In addition, the SRCD spectra of a partially dehydrated film deposited on the surface of a quartz glass plate were collected in the wavelength range of 280–155 nm at 25 °C, with successive rotations on the plate at 0°, 90°, 180°, and 270°.

The oriented SRCD (o-SRCD) of Pln149-PEP20 in lipid bilayers deposited in a circular spot on the surface of a quartz glass Suprasil plate (Hellma Scientific) was measured from 280 nm to 155 nm at 1 nm intervals at 25 °C. Oriented bilayers composed of POPS and POPG were prepared as described by Bürck et al. [61]. The spectra of the bilayers were measured under the same conditions and subtracted from the corresponding sample. All SRCD/o-SRCD spectra were processed using CDToolX [62], by averaging the individual scans, subtracting the respective baseline spectrum, calibrating versus a camphorsulfonic acid standard, zeroing in the 263–270 nm region, smoothing with Savitzky-Golay, and scaling to delta epsilon (Δε) units using a mean amino acid residue weight of 155.4 Da.

### 4.11. Transmission Electron Microscopy

Treated and untreated *S. aureus* ATCC 25923 and *A. baumannii* ATCC 19606 cells were observed by transmission electron microscopy (TEM). Isolated colonies were cultured in CAMH under agitation at 37 °C until the mid-log phase. Then, 30 mL was adjusted to OD_600_ = 0.05 on a SpectraMax M5 (Molecular Devices) in CAMH and treated with the peptide at 0.5×, 1×, or 4× MIC. The tubes were incubated at 37 °C for 10 min, centrifuged three times at 3000× *g* for 10 min, and then washed with PBS. The bacteria were resuspended in PBS with 3% glutaraldehyde and incubated at 4 °C for 2 h, followed by another centrifugation step. Cells were resuspended in PBS and fixed with 3% glutaraldehyde for 2 h at 0 °C, followed by fixation with osmium tetroxide for 2 h at 4 °C. The samples were then washed and dehydrated using increasing concentrations of ethanol. After the final ethanol wash, cells were resuspended in propylene oxide and centrifuged twice. The oxide was removed, and the material was deposited on epoxy resin and stirred overnight. Ultrafine cuts were made using an ultramicrotome. The sections were analyzed using a JEOL 100CX-II microscope (Japan). For quantitative comparison between samples, ten images were captured in random fields at 20,000× magnification. The treated and untreated samples were compared using ANOVA. Bacterial samples were prepared in biological duplicates.

### 4.12. In Vitro Directed Evolution

In vitro directed evolution, guided by the presence of Pln149-PEP20 and antibiotics, was performed in three biological replicates, as described by Jahnsen et al. [60], using *S. aureus* ATCC 25923 and *A. baumannii* ATCC 19606. In addition to ciprofloxacin, daptomycin and polymyxin B were used to treat *S. aureus* and *A. baumannii*, respectively. Initially, the MIC of the peptides or antibiotics had their MIC determined, as described in Section 4.3. On the day of the MIC reading, the absorbance at 600 nm was quantified using a Spectramac M5 (Molecular Devices). In wells where 50% or more inhibition was observed, the content was diluted 1:20 and used as a new bacterial inoculum for a new MIC microplate. This process was repeated daily for 30 days. After 30 days, the final lines were subjected to three passages in CAMH free of antibiotics or peptides for stabilization.

### 4.13. Genome Sequencing

After directed evolution for 30 days, mutants that showed reduced susceptibility to the peptide compared to the initial MIC and the wild-type strains were subjected to DNA extraction for genome sequencing. For the extraction, the QIAGEN^®^ Dneasy Blood and Tissue Kit were used according to the manufacturer’s instructions. According to the manufacturer’s specifications, library preparation for Illumina sequencing was performed using the Nextera XT DNA Library Preparation Kit (Illumina). The quality and quantity of each sample library were measured using a TapeStation instrument (Agilent Technologies). Genomes were sequenced as 2 × 250 bp reads on an Illumina MiSeq sequencer, with a minimum depth of coverage of 126× (ranging from 126× to 242×). Sequence reads were assembled de novo, and variant detection was performed using the CLC Genomics Workbench (CLC Bio, Cambridge, MA). This whole-genome shotgun project has been deposited at DDBJ/ENA/GenBank under the accession numbers JAKSZW000000000, JAKSZX000000000, JAKSZY000000000, JAKWBH000000000, JALLNT000000000, JALLNR000000000, JALLNV000000000, JALLNW000000000, JALLNX000000000, JALLNY000000000, JALLNZ000000000, and JALLKB000000000.

## 5. Conclusions

An optimized analog with biological activity much more relevant than the original Plantaricin 149 was synthesized. Electrostatic interactions play a role in binding this analog to negatively charged lipids, which corresponds with its strong antimicrobial activity against Gram-positive bacteria. However, Pln149-PEP20 was also active against Gram-negative ESKAPE bacteria, and its synergism with vancomycin suggests a potential mode of action on the outer membrane of these organisms. The rapid membrane depolarization suggested that Pln149-PEP20 maintained the Plantaricin 149 carpet mode of membrane interaction.

## Figures and Tables

**Table 1:** Antimicrobial and hemolytic activity of Plantaricin 149 analogs.

Peptide	Sequence	Minimal Inhibitory Concentration (mg/L)								HC_50_ (mg/L)
		*S. epidermidis* ATCC 35984	*S. aureus* ATCC 25923	*E. faecalis* ATCC 29212	*E. faecium* ATCC 700221	*K. pneumoniae* ATCC 700603	*E. coli* ATCC 25922	*A. baumannii* ATCC 19606	*P. aeruginosa* ATCC 27583	
Fmoc-Pln149	Fmoc -YSLQMGATAIKQVKKLFKKKGG	32	256	128	64	256	128	32	256	4
Fmoc-Pln149(6-22)	Fmoc -GATAIKQVKKLFKKKGG	128	512	512	128	512	512	64	512	>128
Pln149-PEP20	Fmoc -KAVKKLFKKWG	4	16	32	16	32	64	32	64	>512

HC_50_ concentration of 50% hemolysis rate.

**Table 2:** Post-antibiotic effect of Pln149-PEP20.

Bacterial Strains	PAE of the Treatments (h ± s.d.)	
	Pln149-PEP20 0.5 × MIC	Pln149-PEP20 1 × MIC
*S. aureus* ATCC 25923 (MIC = 8 mg/L)	4.0 ± 1.0	5.5 ± 2.0
*S. aureus* SA43 [17] (MIC = 8 mg/L)	4.0 ± 0.5	5.0 ± 1.0
*A. baumannii* ATCC 19606 (MIC = 32 mg/L)	3.5 ± 0.5	4.5 ± 0.5
*A. baumannii* ACI50 [18] (MIC = 64 mg/L)	N.O.	2.5 ± 0.5

N.O.: not observed; s.d.: standard deviation.

**Table 3:** Selectivity Index (SI) for Pln149-PEP20.

	CIM_50_ (mg/L)	SI (THP-1)	SI (HFF-1)
Gram-positives	8	8	7
Gram-negatives	32	2	2

**Table 4:** Synergism for Pln149-PEP20 in *A. baumannii* ATCC 19606 (MIC = 32 mg/L).

Antibiotics	Antibiotics MIC (mg/L)	Combination (mg/L)		FIC Index
		MIC_ANTIBIOTIC_	MIC_Pln149-PEP20_	
Ciprofloxacin	1	0.06	8	2
Tobramycin	4	4	16	1.5
Polymyxin B	1	0.25	2	0.312
Vancomycin	>64	2	4	0.133
Ampicillin	>64	16	4	0.375

**Table 5:** Synergism of Gram-negative strains for Pln149-PEP20 and vancomycin.

Bacterial Strains	Main Phenotype	Pln149-PEP20 MIC (mg/L)	Vancomycin MIC (mg/L)	Combination (mg/L)		FIC Index
				MIC_Pln149-PEP20_	MIC_Vancomycin_	
*K. pneumoniae* ATCC 700603		64	>64	8	2	0.133
*K. pneumoniae* AMKP7 [18]	KPC+, CL S	512	128	128	16	0.375
*K. pneumoniae* AMKP4 [18]	KPC+, CL R	512	>64	256	64	0.75
*K. pneumoniae* AMKP10 [18]	KPC+, CL R	512	>64	512	>64	2
*A. baumannii* ATCC 19606		32	>64	4	2	0.133
*A. baumannii* ACI40 [20]	CL S	64	>64	8	2	0.133
*A. baumannii* ACI50 [20]	CL R	64	>64	2	16	0.093
*E. coli* ATCC 25922		32	>64	4	2	0.133
*P. aeruginosa* ATCC 27853	Inducible AmpC	32	>64	32	>64	2

KPC+—*Klebsiella pneumoniae* Carbapenemase (KPC) Producing Bacteria; CL R—Resistant to colistin; CL S—Sensible to colistin.

**Table 6:** Comparison between the initial and final strains of the *S. aureus* ATCC 25923 in vitro selection with Pln149-PEP20.

Replicate	Sequencing Coverage *	N50	Mutation	Change	Altered Protein
A	153×	19,562	G > A	Ala111Thr	Hypothetical protein
			C > T	Ser164Phe	Bifunctional synthetase (p)ppGpp/guanosine-3′-5′-bis(diphosphate)-3′-pyrophosphohydrolase
			A > T	Gln152His	DUF1672 domain protein
			T > A	Ser194Thr	FAD-containing oxidoreductase
B	173×	14,266	394_395Ins **	Frameshift mutation	Fibronectin binding precursor (fnbA)
			C > A	Leu171Ile	Major facilitator superfamily
			5_6Ins ***	Frameshift mutation	Major facilitator superfamily
C	138×	12,146	T > C	Leu893Ser	Clumping factor A (ClfA)
			A > T	Gln152His	DUF1672 domain protein

* Coverages of the initial strains were 148×, 97×, and 112×, respectively; ** Insertion of ACGCTGATGTTGTTGAATATGAA between nucleotides 394 and 395 of the gene encoding the protein; *** Insertion of CT between nucleotides 5 and 6 of the gene encoding the protein.

**Table 7:** Comparison between the initial and final strains of the *A. baumannii* ATCC 19606 in vitro selection with Pln149-PEP20.

Replicate	Sequencing Coverage *	N50	Mutation	Change	Altered Protein
A	201×	14,063	G > C	Ser126Thr	Ig-like domain-containing protein
			A > G	Thr128Ala	
			CT > GC	Thr137Ser	
			A > T	Thr139Ser	
			T > AACC	Asn **	
			AAAT > GGTG	Asn146Val	
			A > C	Lys150Thr	
			TT > CA	Ile154Thr	
			C > A	Pro162Thr	
			GGA > TTG	Gly164Leu	
			CT > GC	Ala168Gly	
			TT > CA	Ile169Thr	
			GAT > TCA	Asp171Ser	
			517_518Ins ***	***	
			T > CACA	Thr ****	
			GACA > TGTG	Thr176Val	
			TA > GG	Asn180Asp	
			A > G	Thr265Ala	
B	190×	8,576	T > AACC	Asn *****	Ig-like domain-containing protein
			AAAT > GGTG	Asn243Val	
			A > C	Lys247Thr	
			TT > CA	Ile251Thr	
			C > A	Pro259Thr	
C	141×	7,603	TA > GG	Asn164Asp	Ig-like domain-containing protein

* Coverages of the initial strains were 173×, 200×, and 178×, respectively; ** Insertion of Asn between Gly144 and Leu145; *** Insertion of ACA between nucleotides 517 and 518 of the gene encoding the protein, resulting in Val173 deletion and insertion of Asp and Ile; **** Insertion of Thr between Val173 and Ala174; ***** Insertion of Asn between Gly241 and Leu242.

## Data Availability

The data presented in this study are available on request from the corresponding author.

## Appendix A

**Table A1:** MIC and MBC of Pln149-PEP20 against Gram-positive bacteria.

Species	Strains	Description	MIC (mg/L)	MBC (mg/L)
*S. epidermidis*	ATCC 35984	Clinical strain, catheter isolate. Strong biofilm former	2	8
	ATCC 12228		1	2
*S. aureus*	ATCC 25923	Clinical strain.	8	16
	SA16 [17]	Clinical strain. MRSA, ST5-SCC*mec*II	8	16
	SA88 *	Clinical strain. MRSA + h-DNSSA, ST5-SCC*mec*II	8	16
	SA43 [17]	Clinical strain. MRSA, ST105- SCC*mec*II, TIG S	8	8
	SA43 B2 [17]	In vitro selected MRSA, ST105- SCC*mec*II, TIG S	8	8
	SA43 B7 [17]	In vitro selected ST105- MRSA, SCC*mec*II, TIG R (*mepR* mutation)	8	8
	Mu50 [52]	MRSA, VISA, ST5	2	>8
	ATCC 8095	Food isolate. Strong biofilm former	8	16
*E. faecalis*	VRE 109 [53]	Clinical strain, ST103, *vanA* TIG S, VAN R	32	128
	VRE 109 42C [53]	In vitro selected. ST103 *vanA* TIG R, VAN R	32	128
	VRE 80 [53]	Clinical strain. ST103 *vanA* TIG R, VAN R,	32	128
	V583	ST 6, *vanB.* VAN R, CN R	128	256
	RPEfs1 *	Clinical strain. AMP S, CIP R, ERY R, LNZ S, MXF R, TEC S, TIG S, VAN S	128	128
	RPEfs2 *	Clinical strain. AMP S, CIP S, ERY I, LNZ S, MXF S, TEC S, TIG S, VAN S	128	128
	RPEfs3 *	Clinical strain. AMP S, CIP R, ERY R, LNZ S, MXF R, TEC R, TIG S, VAN R	128	128
	RPEfs4 *	Clinical strain. AMP S, CIP R, ERY R, LNZ S, MXF S, TEC S, TIG S, VAN S	8	32
	RPEfs5 *	Clinical strain. AMP S, CIP S, DAP S, LNZ S, NIT S, TEC S, TET R, VAN S	8	>32
	ATCC 29212	Clinical strain, urine isolate	32	64
*E. faecium*	VRE 16 [54]	Clinical strain. ST412, *vanA.* VAN R	32	32
	HBSJRP18 [55]	Clinical strain. ST412, DAP supersusceptible (*lafB* *)	32	64
	HBSJRP18 2.7 [55]	In vitro selected. DAP S	32	64
	HBSJRP18 3.6 [55]	In vitro selected. DAP R (*dak **)	32	32
	HBSJRP7 [55]	Clinical strain, muscle biopsy isolate ST896, *ermB, msrC, tetL, tetM, vanA*. DAP R, LNZ S, TED S, TEC R, VAN R	16	32
	HBSJRP13 [55]	Clinical strain, bronchus alveolar lavage isolate ST896, *ermB, msrC, tetL, tetM, vanA.* DAP S, LNZ S, TED S, TEC R, VAN R	16	32
	HBSJRP14 [55]	Clinical strain, urine isolate. *vanA.* DAP S, LNZ S, TED S, TEC R, VAN R	16	64
	HBSJRP23 [55]	Clinical strain, urine isolate. DAP S, LNZ S, TED S, TEC S, VAN S	8	8
	HBSJRP11 [55]	Clinical strain, urine isolate DAP S, LNZ S, TED S, TEC S, VAN S	8	16
	ATCC 700221	Human feces isolate. *vanA*, VAN R	8	32

* Ilana Camargo, LEMiMo. American Type Culture Collection (ATCC), Sequence type (ST), Contains vancomycin resistance element VanA (vanA), methicillin-resistant *Staphylococcus aureus* (MRSA), S. aureus with intermediate vancomycin resistance (VISA), resistant (R), sensitive (S), intermediate (I), Amikacin (AK), Amoxicillin-clavulanate (AMC), Ampicillin (AMP), Ampicillin-sulbactam (SAM), Aztreonam (ATM), Cefepime (FEP), Cefotaxime (CTX), Cefoxitin (FOX), Cefpodoxime (CPD), Ceftazidime (CAZ), Ceftriaxone (CRO), Cefuroxime (CXM), Ciprofloxacin (CIP), Chloramphenicol (CHL), Colistin (CL), Daptomycin (DAP), Erythromycin (ERY), Ertapenem (ETP), Gentamicin (CN), Imipenem (IMI), Linezolid (LNZ), Meropenem (MEM), Moxifloxacin (MXF), Nitrofurantoin (NIT), Piperacillin (PIP), Piperacillin-tazobactam (PTZ), Polymyxin B (PB), Tedizolide (TED), Teicoplanin (TEC), Tetracycline (TET), Tigecycline (TIG), Trimethoprim-sulfamethoxazole (SXT), Vancomycin (VAN).

**Table A2:** MIC and MBC of Pln149-PEP20 against Gram-negative bacteria.

Species	Strains	Description	MIC (mg/L)	MBC (mg/L)
*K. pneumoniae*	ATCC 700603	Clinical strain, urine isolate. *bla*_KPC_-, *bla*_SHV-18_ + AMP R, ATM R, FOX R, CPD R, CAZ R, CHL R, PIP R, TET R	16	>64
	ATCC BAA1705	Clinical strain, urine isolate. *bla*_KPC_+	16	>64
	BHKPC50 *	Clinical strain, urine isolate. AK R, AMP R, SAM R, FEP R, FOX R, CAZ R, CRO R, CXM R, CIP R, CL R, ETP R, CN R, IMI R, MEM R, PTZ R, TIG R	128	512
	RPKp01 *	Clinical strain, urine isolate. AK S, AMP S, SAM R, FEP R, FOX R, CAZ R, CRO R, CXM R, CIP R, CL S, ETP R, CN S, IMI R, MEM R, PTZ R, TIG R	32	64
	RPKp02 *	Clinical strain, rectal swab isolate. AK I, AMP R, SAM R, FEP R, FOX R, CAZ R, CRO R, CXM R, CIP R, CL R, ETP R, CN S, IMI R, MEM R, PTZ R, TIG I	128	256
	RPKp09 *	Clinical strain, surgical wound isolate. AK S, AMC R, AMP R, FEP R, FOX R, CAZ R, CRO R, CXM R, CIP S, CL S, ETP S, CN S, IMI R, MEM R, PTZ R, TIG R,	32	64
	RPKp18 *	Clinical strain, blood isolate. AK S, AMP R, SAM R, FEP R, FOX R, CAZ R, CRO R, CXM R, CIP S, CL R, ETP R, CN S, IMI R, MEM R, PTZ R, TIG S	128	256
	NDM-1 (2146) **	NDM +	32	>128
	AMKP4 [18]	Clinical strain. *bla*_KPC-2_ CL R, ETP R, IMI R, MEM R, PB R, TIG S	512	>512
	AMKP7 [18]	Clinical strain. *bla*_KPC-2_ CL S, ETP R, IMI R, MEM R, PB S, TIG S	256	256
	AMKP10 [18]	Clinical strain. ST2306, *bla*_KPC-2_, *bla*_CTX-M8_, *bla*_SHV-11_, *tetA, aph(3′)-Ia, catB, aac(6′)Ib-cr, fosA, bla*_CTX-M15_, *oqxab, qnrS1, sul1, bla*_OXA-1_, *aadA2, dfrA12, mph(A), mgr*B AK S, AMP R, SAM R, FEP R, FOX R, CAZ R, CRO R, CXM R, CIP R, CL R, ETP R, CN S, IMI R, MEM R, PB R, TIG S	512	>512
*E. coli*	ATCC 25922		32	64
	ATCC 35218	Canine isolate. TEM-1 +	32	128
	RPEc01 *	Clinical strain, urine isolate. AK R, AMP R, SAM R, FEP R, FOX R, CAZ R, CRO R, CXM R, CIP S, CL S, ETP R, CN R, IMI R, MEM R, PTZ R, TIG S	32	128
	BHKPC10 *	Clinical strain, urine isolate. AK S, AMC R, FEP R, CAZ R, CIP S, CN S, MEM R, NIT S, SXT S.	32	128
	AMEc8 *	Clinical strain, urine isolate. AK S, AMP R, SAM R, FEP R, FOX R, CAZ R, CRO R, CXM R, CIP R, CL S, ETP R, CN R, IMI S, MEM S, PTZ R, TIG I	16	32
	AMEc49 *	Clinical strain, cranial subdural empyema isolate. AK S, AMP R, SAM I, FEP S, FOX S, CAZ S, CRO R, CXM R, CIP S, CL S, MEM S, PTZ S, TIG S	32	64
	AMEc60 *	Clinical strain, tracheal secretion isolate. AK S, AMP R, SAM R, FEP R, FOX S, CAZ R, CRO R, CXM R, CIP R, CL S, MEM S, PTZ S, TIG S	16	64
*A. baumannii*	ATCC 19606	Clinical strain, urine isolate.	32	64
	ACI50 [20]	Clinical strain. AK R, SAM R, FEP R, CTX R, CAZ R, CRO R, CIP R, CL R, CN R, IMI R, MEM R, PTZ R PB R, TET I, TIG R, SXT R	64	128
	ACI44 [20]	Clinical strain. AK R, SAM R, FEP R, CTX R, CAZ R, CRO R, CIP R, CL S, CN R, IMI R, MEM R, PTZ R, PB S, TET R, TIG R, SXT R	128	256
	ACI51 [20]	Clinical strain. AK R, SAM R, FEP R, CTX R, CAZ R, CRO R, CIP R, CL R, CN R, IMI R, MEM R, PTZ R PB R, TET I, TIG R, SXT R	256	256
	ACI40 [20]	Clinical strain. AK R, SAM R, FEP R, CTX R, CAZ R, CRO R, CIP R, CL S, CN R, IMI R, MEM R, PTZ R, PB S, TET R, TIG R, SXT R	64	128
	ACI42 [20]	Clinical strain. AK R, SAM R, FEP R, CTX R, CAZ R, CRO R, CIP R, CL S, CN R, IMI R, MEM R, PTZ R, PB S, TET R, TIG R, SXT R	64	256
	AM83 *	Clinical strain, tracheal secretion isolate. AK R, SAM R, FEP R, CAZ R, CRO R, CL S	32	128
	AM87 *	Clinical strain, urine isolate. AK S, SAM R, FEP R, CAZ R, CRO R, CIP R, CL S	16	16
*P. aeruginosa*	ATCC 27853	Clinical strain, blood isolate. Inducible AmpC	32	128
	RPPse09 *	Clinical strain, rectal swab isolate. AK S, FEP S, CAZ S, CIP S, CL S, CN S, IMI R, MEM R, PTZ S	256	512
	RPPse07 *	Clinical strain, urine isolate. AK R, FEP R, CAZ I, CIP R, CL S, CN R, IMI R, MEM R, PTZ I	64	128
	PSE6 [56]	KPC+	128	128
	PAO1		128	256

* Ilana Camargo, LEMiMo, ** Ana Cristina Gales (LEMC/Alerta), American Type Culture Collection (ATCC), Sequence type (ST), resistant (R), sensitive (S), intermediate (I); Amikacin (AK), Amoxicillin-clavulanate (AMC), Ampicillin (AMP), Ampicillin-sulbactam (SAM), Aztreonam (ATM), Cefepime (FEP), Cefotaxime (CTX), Cefoxitin (FOX), Cefpodoxime (CPD), Ceftazidime (CAZ), Ceftriaxone (CRO), Cefuroxime (CXM), Ciprofloxacin (CIP), Chloramphenicol (CHL), Colistin (CL), Daptomycin (DAP), Erythromycin (ERY), Ertapenem (ETP), Gentamicin (CN), Imipenem (IMI), Linezolid (LNZ), Meropenem (MEM), Moxifloxacin (MXF), Nitrofurantoin (NIT), Piperacillin (PIP), Piperacillin-tazobactam (PTZ), Polymyxin B (PB), Tedizolide (TED), Teicoplanin (TEC), Tetracycline (TET), Tigecycline (TIG), Trimethoprim-sulfamethoxazole (SXT), Vancomycin (VAN).
